# Bacillus anthracis Responds to Targocil-Induced Envelope Damage through EdsRS Activation of Cardiolipin Synthesis

**DOI:** 10.1128/mBio.03375-19

**Published:** 2020-03-31

**Authors:** Clare L. Laut, William J. Perry, Alexander L. Metzger, Andy Weiss, Devin L. Stauff, Suzanne Walker, Richard M. Caprioli, Eric P. Skaar

**Affiliations:** a Department of Pathology, Microbiology, and Immunology, Vanderbilt University Medical Center, Nashville, Tennessee, USA; b Vanderbilt Institute for Infection, Immunology, and Inflammation, Vanderbilt University Medical Center, Nashville, Tennessee, USA; c Mass Spectrometry Research Center, Vanderbilt University, Nashville, Tennessee, USA; d Department of Chemistry, Vanderbilt University, Nashville, Tennessee, USA; e Department of Biology, Grove City College, Grove City, Pennsylvania, USA; f Department of Microbiology and Molecular Genetics, Harvard Medical School, Boston, Massachusetts, USA; g Department of Biochemistry, Vanderbilt University, Nashville, Tennessee, USA; h Department of Pharmacology, Vanderbilt University, Nashville, Tennessee, USA; i Department of Medicine, Vanderbilt University Medical Center, Nashville, Tennessee, USA; New York University School of Medicine

**Keywords:** *Bacillus anthracis*, antimicrobial agents, cell envelope, phospholipids, two-component regulatory systems

## Abstract

Bacillus anthracis is a spore-forming bacterium that causes devastating infections and has been used as a bioterror agent. This pathogen can survive hostile environments through the signaling activity of two-component systems, which couple environmental sensing with transcriptional activation to initiate a coordinated response to stress. In this work, we describe the identification of a two-component system, EdsRS, which mediates the B. anthracis response to the antimicrobial compound targocil. Targocil is a cell envelope-targeting compound that is toxic to B. anthracis at high concentrations. Exposure to targocil causes damage to the cellular barrier and activates EdsRS to induce expression of a previously uncharacterized cardiolipin synthase, which we have named ClsT. Both EdsRS and ClsT are required for protection against targocil-dependent damage. Induction of *clsT* by EdsRS during targocil treatment results in an increase in cardiolipin levels, which protects B. anthracis from envelope damage. Together, these results reveal that a two-component system signaling response to an envelope-targeting antimicrobial induces production of a phospholipid associated with stabilization of the membrane. Cardiolipin is then used to repair envelope damage and promote B. anthracis viability.

## Abstract (precis)

Compromising the integrity of the bacterial cell barrier is a common action of antimicrobials. Targocil is an antimicrobial that is active against the bacterial envelope. We hypothesized that Bacillus anthracis, a potential weapon of bioterror, senses and responds to targocil to alleviate targocil-dependent cell damage. Here, we show that targocil treatment increases the permeability of the cellular envelope and is particularly toxic to B. anthracis spores during outgrowth. In vegetative cells, two-component system signaling through EdsRS is activated by targocil. This results in an increase in the production of cardiolipin via a cardiolipin synthase, ClsT, which restores the loss of barrier function, thereby reducing the effectiveness of targocil. By elucidating the B. anthracis response to targocil, we have uncovered an intrinsic mechanism that this pathogen employs to resist toxicity and have revealed therapeutic targets that are important for bacterial defense against structural damage.

## INTRODUCTION

Bacillus anthracis is among the few organisms that have been used as a bioterror weapon (1–4). Four infectious syndromes can result from exposure to B. anthracis spores: cutaneous, gastrointestinal, inhalation, and injectional anthrax (1, 3, 5–7). Inhalation of anthrax results in the most severe disease, with mortality rates approaching 90% (8). Upon exposure to host tissues, spores are phagocytosed by immune cells in an attempt to eliminate the pathogen. Spores that survive phagocytic attack germinate into vegetative cells that avoid immune-mediated clearance and cause life-threatening disease. Accordingly, B. anthracis is well equipped to respond to a range of stressors experienced during vertebrate colonization.

The infectivity of bacteria that colonize mammals, such as B. anthracis, is dependent upon the ability to sense and respond to the host environment. Pathogens can rapidly alter gene expression in response to environmental stimuli using two-component systems (TCSs). TCSs typically consist of a membrane-embedded sensor histidine kinase and cognate response regulator. The sensor kinase becomes activated upon exposure to a specific signal(s), resulting in autophosphorylation. The kinase activates the regulator via phosphotransfer, and the regulator, in turn, commonly acts as a transcription factor, resulting in gene expression changes to support survival and growth. Although genes encoding TCSs are often readily identifiable, the activating signals for TCS are not well defined. Some TCSs, such as the TCS PhoPQ, respond to multiple signals. This TCS is conserved between Gram-positive and Gram-negative species and can be activated by antimicrobial peptides or altered metal levels to provide resistance to antimicrobial peptides (9–12). Others, like Staphylococcus aureus AgrCA, are activated by binding a single ligand such as the quorum sensing molecule autoinducing peptide (AIP) (13). Disruption of the phospholipid membrane can be sensed by TCSs. For instance, DesKR in B. subtilis is a thermosensor that is activated at low temperatures when the cellular membrane condenses to reveal the linker domain in the sensor kinase and promote autokinase activity (14–22). Activation of DesKR results in the synthesis of unsaturated fatty acids that maintain membrane fluidity under colder conditions (14). These are some of the few known examples of TCSs with a defined stimulus and response pattern that is vital for the stability of the cell.

In Gram-positive species, the cell is protected by a single phospholipid bilayer and the peptidoglycan cell wall (23, 24). In some cases, there is an additional proteinaceous S-layer and an antiphagocytic capsule (25–27). Maintenance of an intact cell envelope is required for bacterial survival, including during growth within vertebrates. The bacterial cell envelope provides protection from environmental assaults to maintain redox state, preserve nutrient pools, and defend against antimicrobial attack, among other activities. Targocil is an example of an antibacterial that inhibits elaboration of wall teichoic acid (WTA) in S. aureus. Targocil inhibits TarG, the permease component of the ATP binding cassette (ABC) transporter responsible for the export of fully synthesized WTA to the surface of S. aureus (28). However, not all Gram-positive pathogens synthesize WTA; one WTA-negative species is B. anthracis (29, 30). These findings prompted the hypothesis that targocil activity against B. anthracis must occur in a WTA-independent manner.

In this study, we demonstrated that targocil treatment of B. anthracis activates a previously unstudied TCS, EdsRS. Upon activation, EdsRS upregulates self-expression and expression of an additional operon, consisting of *BAS1661-BAS1663clsT.* Elevated expression of *clsT* is required for the protection of B. anthracis against alterations in barrier permeability caused by targocil treatment, which are heightened in defined media and during spore germination. *clsT* encodes a newly identified cardiolipin synthase that is responsible for increasing the abundance of cardiolipin and thus restoring envelope damage caused by targocil. This work describes how antimicrobial-mediated activation of a two-component system in B. anthracis initiates a membrane remodeling response to maintain bacterial fitness.

## RESULTS

### Bacillus anthracis transcriptional responses to the antibacterial compound targocil.

**FIG 1:**
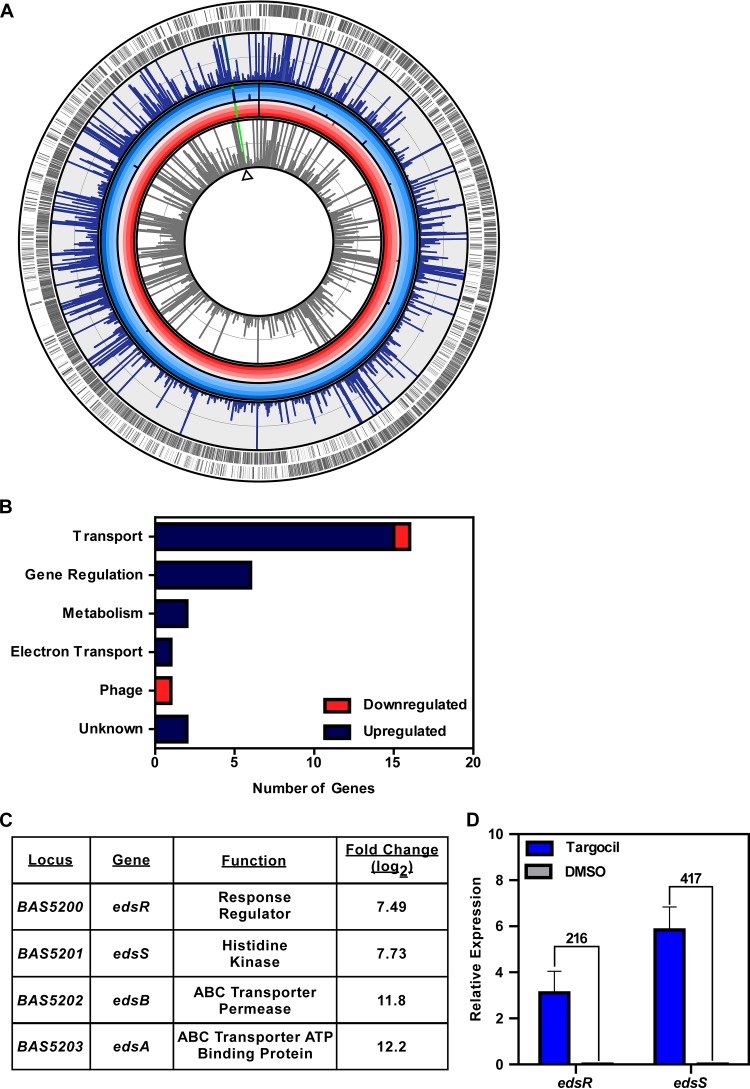
Targocil induces gene expression changes in B. anthracis. (A) Genomic map of B. anthracis Sterne. Indicated are the open reading frames (outer ring in gray) and on which strand the gene is present. The degree of expression for each transcript is shown for cultures grown in targocil (blue bars in outer ring) and DMSO control (gray bars). Those transcripts which were significantly altered with a log_2_ fold change value of at least 2 and the degree of fold change in increments of 3 (concentric gradient) are shown for transcripts that were upregulated (navy bars) and downregulated (red bars). The most significantly upregulated operon is indicated (green bar). (B) The number of transcripts that fall into unique KEGG pathways are displayed, as well as whether they were upregulated or downregulated. (C) Fold change values for those transcripts that were most significantly increased in abundance after targocil treatment. Included are the locus, gene name, predicted protein function, and log_2_ fold change. (D) qRT-PCR validation of transcriptome sequencing (RNA-Seq) results for the *edsRS* operon represented by targocil-induced expression relative to expression in DMSO. Fold changes between targocil-treated and DMSO-treated samples are shown above the relative expression bars. Data represent averages of results from three independent experiments performed in biological triplicate ± standard errors of the means (SEM).

The recently identified antibiotic targocil inhibits WTA biosynthesis in S. aureus. To identify the mechanism of action of targocil in B. anthracis, which lacks WTA, we defined the transcriptional response of B. anthracis to targocil by RNA sequencing. Alterations in the gene expression profile of the parent B. anthracis strain after a brief exposure to targocil were measured during mid-exponential-phase growth in rich medium. The attenuated B. anthracis Sterne strain (31) served as the parent strain in all experiments in this study. There were 28 total transcripts significantly altered in targocil-treated samples, the majority of which were upregulated (Fig. 1A; see also Table S1, Table S2, Table S3, and Table S4 in the supplemental material). The primary pathways affected by targocil included transport and gene regulation, though transcriptional changes were also observed in genes involved in metabolism and electron transport and in phage genes (Fig. 1B). One set of genes, *BAS5200* to *BAS5203*, displayed the highest fold change compared to untreated cells. Alignment of the sequencing reads to the genome suggests that genes *BAS5200* to *BAS5203* are in an operon and are expressed as a monocistronic transcript. This operon codes for a putative response regulator (*BAS5200*), a histidine kinase (*BAS5201*), an ABC transporter permease (*BAS5202*), and an ABC transporter ATP-binding protein (*BAS5203*). BAS5200 and BAS5201 contain domains associated with known two-component systems. BAS5201 is predicted to contain a HisKA3 phosphoacceptor domain and a HATPase domain for ATP hydrolysis (32, 33). BAS5200 contains a phosphoacceptor domain for activation by the histidine kinase and a GerE helix-turn-helix domain for DNA binding (34). *BAS5202-BAS5203* codes for a putative ABC transporter. This family of proteins is associated with the transport of a wide range of substrates, including antimicrobials (35). Due to the described role of targocil in disrupting barrier function, and for reasons described below, we named this operon the envelope disruption sensor (*eds*) system (*edsRSAB*) (Fig. 1C). Quantitative reverse transcription-PCR (qRT-PCR) was performed on transcripts isolated from targocil-treated samples to confirm RNA sequencing results. Treatment of the parent strain with targocil showed levels of expression of both *edsR* and *edsS* that were higher than those seen with untreated samples (Fig. 1D). Collectively, the findings suggest that targocil treatment induces expression of a putative TCS (*edsRS*) and an ABC transporter (*edsAB*).

10.1128/mBio.03375-19.4 TABLE S1 Parental targocil versus parental DMSO. Download Table S1, DOCX file, 0.01 MB. Copyright © 2020 Laut et al. 2020 Laut et al. This content is distributed under the terms of the Creative Commons Attribution 4.0 International license.

10.1128/mBio.03375-19.5 TABLE S2 Δ*edsRS* targocil versus Δ*edsRS* DMSO. Download Table S2, DOCX file, 0.02 MB. Copyright © 2020 Laut et al. 2020 Laut et al. This content is distributed under the terms of the Creative Commons Attribution 4.0 International license.

10.1128/mBio.03375-19.6 TABLE S3 Δ*edsRS* targocil versus parental targocil. Download Table S3, DOCX file, 0.01 MB. Copyright © 2020 Laut et al. 2020 Laut et al. This content is distributed under the terms of the Creative Commons Attribution 4.0 International license.

10.1128/mBio.03375-19.7 TABLE S4 Δ*edsRS* DMSO versus parental DMSO. Download Table S4, DOCX file, 0.01 MB. Copyright © 2020 Laut et al. 2020 Laut et al. This content is distributed under the terms of the Creative Commons Attribution 4.0 International license.

### EdsRS signaling is activated in response to targocil.

**FIG 2:**
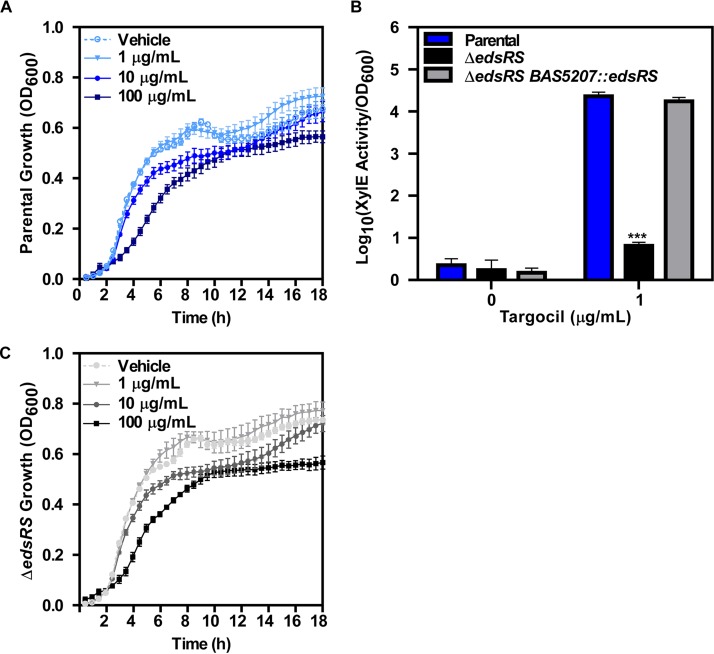
Targocil induces expression of *edsRSAB* in an EdsRS-dependent manner. (A) Growth of B. anthracis Sterne (parental strain) measured by optical density at 600 nm (OD_600_) in LB containing 0, 1, 10, or 100 μg/ml targocil. Data represent averages of results from two independent experiments performed in biological triplicate ± SEM. (B) Activity of *eds* promoter in response to targocil was quantified using parental, Δ*edsRS*, and Δ*edsRS BAS5207*::*edsRS* strains carrying the *xylE* reporter plasmid. Strains were grown in triplicate in the presence of vehicle or 1 μg/ml of targocil for 6 h. XylE activity was quantified and normalized to bacterial density at the time of assay termination. Data represent averages of results from three independent experiments performed in biological triplicate ± SEM. Statistical significance compared to parental strain results was determined using a two-way analysis of variance (ANOVA) with a Tukey’s test adjustment for multiple comparisons (***, *P ≤ *0.001). (C) Growth of the Δ*edsRS* strain measured by OD_600_ in LB containing 0, 1, 10, or 100 μg/ml targocil. Data represent averages of results from two independent experiments performed in biological triplicate ± SEM.

Targocil at high concentrations is toxic to B. anthracis grown in rich media (Fig. 2A). However, the resistance of B. anthracis to targocil is over 100-fold higher than the level previously observed in S. aureus (36). We hypothesized that the EdsRS two-component system is activated in B. anthracis to resist targocil-dependent killing. This was quantified using a vector that contained the *eds* promoter driving expression of a catechol oxidase, XylE. Activation of the promoter can be measured by quantifying the rate of conversion of catechol to a colorimetric product using a spectrophotometer (37). In the parent strain, targocil treatment increased P*_eds_* activation compared to a vehicle control (Fig. 2B). The Δ*edsRS* mutant lacks this activation, and this phenotype can be complemented by expressing *edsRS* in *trans* (Δ*edsRS BAS5207*::*edsRS*) (Fig. 2B). These results demonstrate that targocil exposure activates *edsRSAB* expression and that this is dependent on EdsRS. However, when the parent strain and Δ*edsRS* were grown in rich medium containing increasing concentrations of targocil, increased susceptibility of Δ*edsRS* to targocil was not observed (Fig. 2). These data suggest that targocil-dependent activation of EdsRS signaling induces expression of its own operon, but that this TCS-activation is not required for resistance to toxicity. We propose that this could be due to the presence of additional mechanisms that can defend against targocil-induced damage and are active in mid-log cultures. EdsRS is activated under these conditions, but alternative systems could compensate in maintenance of cellular stability.

### Targocil activates EdsRS-dependent expression of *BAS1661-BAS1663clsT*.

**FIG 3:**
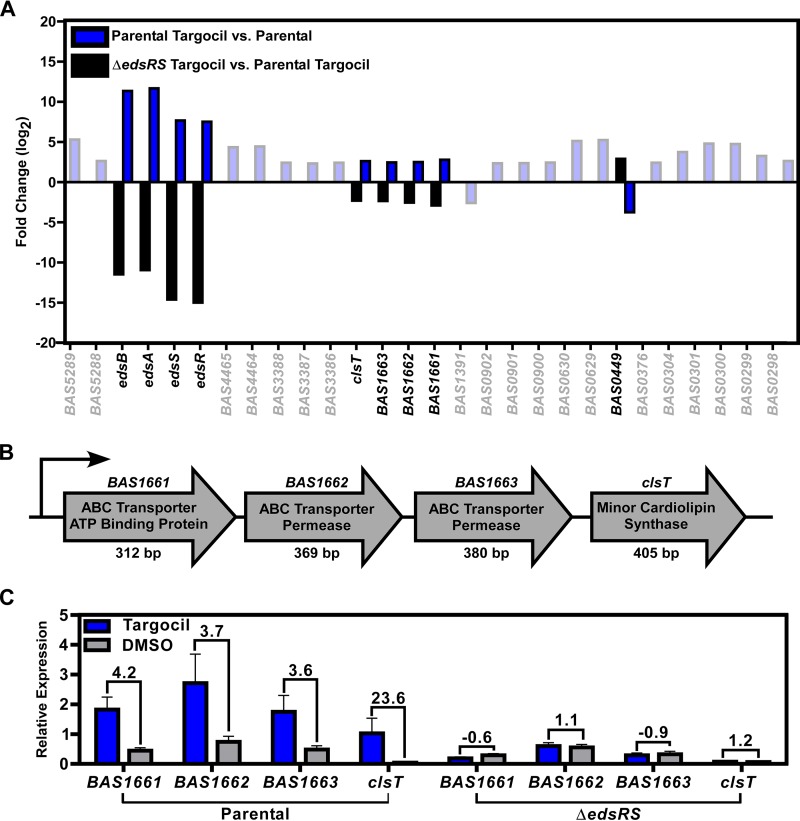
Targocil activation of EdsRS leads to induction of *BAS1661-BAS1664* expression. (A) Depiction of the significant fold changes for two distinct RNA sequencing comparisons. Shown are those genes altered in B. anthracis Sterne (parental) treated with targocil compared to the parental strain left untreated (blue) and the Δ*edsRS* mutant treated with targocil compared to the parental strain treated with targocil (black). Data for transcripts shown as having been altered under the same conditions but in the opposite direction indicate genes altered by targocil that require *edsRS* (dark bars). (B) *BAS1661* to *BAS1663clsT* encode an ABC transporter ATP binding protein (*BAS1661*), an ABC transporter permease (*BAS1662*), an ABC transporter permease (*BAS1663*), and a cardiolipin synthase (*clsT*). (C) qRT-PCR validation represented by relative levels of expression of the *BAS1661-BAS1663clsT* operon in the parental strain and a Δ*edsRS* mutant under conditions of growth in DMSO and targocil. Data represent averages of results from three independent experiments performed in biological triplicate ± SEM. Fold changes between targocil-treated and DMSO-treated samples are shown above the relative expression bars.

To define the regulon of EdsRS, we performed RNA sequencing on the parent strain and the Δ*edsRS* mutant, which were treated with or without targocil. Targocil and EdsRS are required for expression changes of *edsRSAB* and of an uncharacterized operon, *BAS1661* to *BAS1664.* Both operons were upregulated in the presence of targocil, in an EdsRS-dependent manner (Fig. 3A). Alignment of the RNA sequencing reads to the region of *BAS1661* to *BAS1664* suggests that these genes are expressed as an operon. This operon contains putative ABC transporter genes (*BAS1661-BAS1663*) and a putative cardiolipin synthase gene (*BAS1664*) (Fig. 3B). BAS1661-BAS1663 are predicted to contain domains from the ABC-2 subfamily of transporters associated with the export of drugs and carbohydrates (38). In addition to *BAS1664*, the genome of B. anthracis Sterne includes four other putative cardiolipin synthase genes (see Fig. S1A in the supplemental material). BAS1664 contains the HKD motif associated with this superfamily of proteins that is required for the synthesis of cardiolipin (39, 40). There is significant protein identity shared between the previously described ClsA and ClsB proteins of B. subtilis and BAS1112 and BAS5195, respectively, but BAS1664 shares less identity with the previously characterized proteins (Fig. S1B). On the basis of the activation in response to targocil, we have named *BAS1664* cardiolipin synthase targocil (*clsT*). To confirm that EdsRS activation results in upregulation of *BAS1661-BAS1663clsT*, we performed qRT-PCR on RNA extracted from the parent strain and the Δ*edsRS* mutant, either treated with targocil or left untreated (Fig. 3C). These results indicate that targocil activates EdsRS to increase expression of *BAS1661-BAS1663clsT*. This suggests that ClsT is a previously unstudied cardiolipin synthase that is expressed when EdsRS is activated by targocil.

10.1128/mBio.03375-19.1 FIG S1 (A) Genetic map of cardiolipin synthase genes in B. anthracis. Shown in purple are the cardiolipin synthase genes, with *clsT* shown in dark purple. Clustal Omega was used to calculate the percentage of protein sequence identity of each cardiolipin synthase compared to ClsT. This value is represented below each gene. The genetic context of each synthase gene is indicated with gray arrows. (B) Heat map representing the percentage of protein sequence identity of the B. anthracis predicted cardiolipin synthase genes compared to the cardiolipin synthase genes referenced in this study. Percent identity was obtained through the comparison of sequences using the Clustal Omega Multiple Sequence Alignment tool (98). (C) The protein sequences of B. anthracis ClsT and other cardiolipin synthase proteins discussed in this study are fully aligned. Regions of continuous homology are represented by a line, with the overall level of conservation at each residue represented via heat map data. The ClsT catalytic HKD domain is aligned with those of the described cardiolipin synthase proteins, and the level of conservation is shown. B. anthracis codes for one HKD domain, while all other sequences analyzed contained two of the characteristic H-x-K-x(4)-D-x(6)-G-x-x-N motifs. Download FIG S1, TIF file, 2.3 MB. Copyright © 2020 Laut et al. 2020 Laut et al. This content is distributed under the terms of the Creative Commons Attribution 4.0 International license.

### EdsRS signaling is required for spore outgrowth in the presence of targocil.

**FIG 4:**
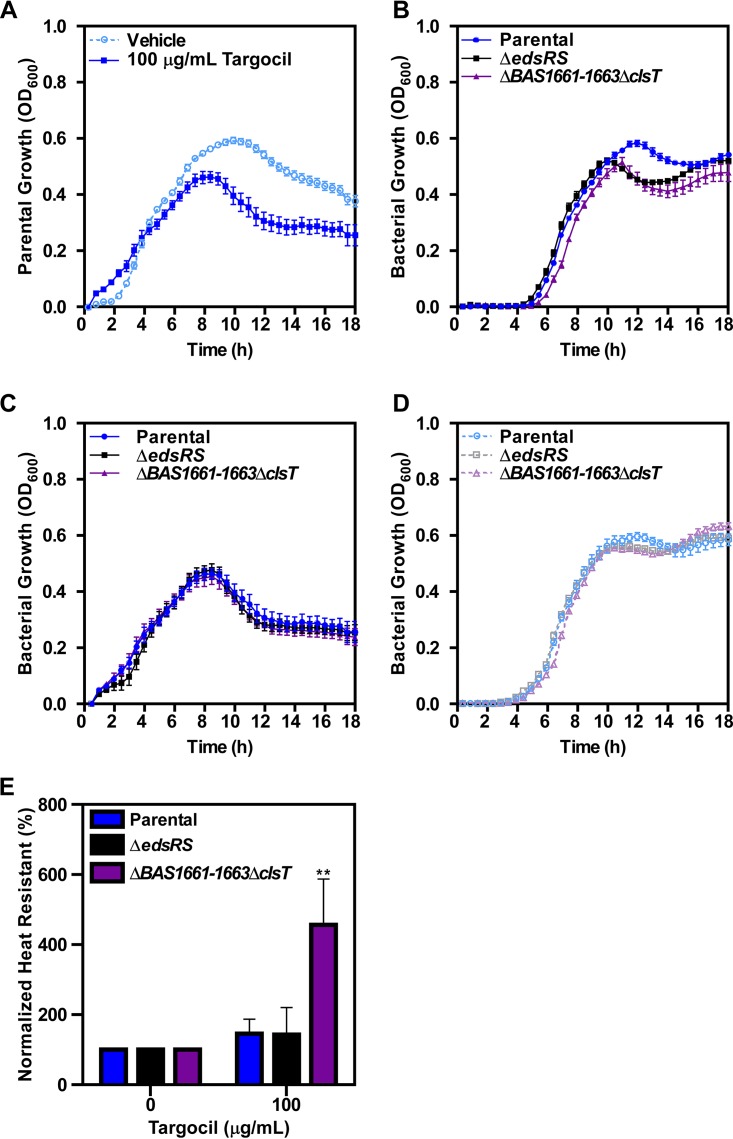
Activation of EdsRS signaling is required for spore germination during targocil treatment in defined media. (A) Growth of B. anthracis Sterne (parental strain) measured by OD_600_ in RPMI medium plus 1% Casamino Acids containing 0 or 100 μg/ml targocil. Data represent averages of results from three independent experiments performed in biological quadruplicate ± SEM. (B) Germination and subsequent growth of parental strain, Δ*edsRS* mutant, and Δ*BAS1661-BAS1663*Δ*clsT* mutant spores in RPMI medium plus 1% Casamino Acids containing 25 μg/ml targocil. Data represent averages of results from three independent experiments performed in biological triplicate ± SEM. (C) Growth of the parental, Δ*edsRS*, *and* Δ*BAS1661-BAS1663*Δ*clsT* strains measured by OD_600_ in RPMI medium plus 1% Casamino Acids containing 100 μg/ml targocil. Data represent averages of results from three independent experiments performed in biological quadruplicate ± SEM. (D) Germination and subsequent growth of the parental, Δ*edsRS*, and Δ*BAS1661-BAS1663*Δ*clsT* spores in RPMI medium plus 1% Casamino Acids containing a vehicle control. Data represent averages of results from three independent experiments performed in biological triplicate ± SEM. (E) After 5 min of incubation of the parental, Δ*edsRS*, and Δ*BAS1661-BAS1663*Δ*clsT* spores with rich media with and without 100 μg/ml targocil, samples were analyzed for total bacterial counts and heat resistance. The percentage of heat-resistant bacteria is normalized to the control treated sample. Data presented are the averages of three independent experiments performed in biological triplicate ± SEM. Statistical significance compared to other treated samples was determined using a two-way ANOVA with a Tukey’s test adjustment for multiple comparisons (**, *P ≤ *0.01).

Targocil exposure inhibits growth of B. anthracis. On the basis of the EdsRS-dependent activation of ClsT, we hypothesized that EdsRS senses targocil to coordinate the increase in barrier stability required for growth during treatment. To test this hypothesis under defined conditions, growth experiments were performed in RPMI medium with Casamino Acids (41–44). Growth of the parent strain during targocil treatment in RPMI medium was assessed by measuring optical density at 600 nm (OD_600_) over time. Cultures grown in 100 μg/ml targocil did not reach the same maximum optical density as those grown in the presence of vehicle control, indicating that targocil is toxic in this semidefined media (Fig. 4A).

The infectious particle of B. anthracis is the endospore, which germinates during infection of a mammalian host (1, 3, 45–48). As a spore germinates, the cell must elongate into a full-length vegetative cell. This process involves the elaboration of cellular barriers, including the phospholipid bilayer, S-layer, and peptidoglycan wall. We hypothesized that targocil would be more toxic to newly germinating B. anthracis spores than to actively growing vegetative cells. To test this, complete growth kinetics after spores were inoculated into RPMI medium containing targocil were assessed. Outgrowth of spores lacking Δ*BAS1661-BAS1663*Δ*clsT* showed a slight growth delay compared to the parental spores in the presence of targocil (Fig. 4B). Additionally, both the Δ*BAS1661-BAS1663*Δ*clsT* spores and the Δ*edsRS* spores exhibited altered growth kinetics at later time points in stationary phase upon targocil exposure. These phenotypes were not observed when exponential-phase cultures were used for inoculation to determine growth curves under the same conditions (Fig. 4C). The mutant strains did not display altered growth compared to the parental strain in semidefined medium containing a vehicle control (Fig. 4D). This indicates that the Δ*BAS1661-BAS1663*Δ*clsT* spores and the Δ*edsRS* spores were not less fit overall and that the altered growth was due to the activity of targocil. We hypothesized that the defect in outgrowth observed in the Δ*BAS1661-BAS1663*Δ*clsT* spores and the Δ*edsRS* spores (Fig. 4B) was due to delayed spore germination in the presence of targocil. An indicator of spore germination is the loss of heat resistance. The degree of heat resistance that remained in samples exposed to rich media in the presence of vehicle or targocil was measured. Germination of the Δ*BAS1661-BAS1663*Δ*clsT* strain was significantly reduced in samples treated with targocil compared to all other conditions (Fig. 4E). Disc diffusion assays were performed using the parent strain and the Δ*edsRS* and Δ*BAS1661-BAS1663*Δ*clsT* strains challenged with a panel of toxic compounds. Of those tested, no additional compounds were found to have differential toxicity in the mutant strains compared to the parent strain (Fig. S2). Therefore, EdsRS, BAS1661-BAS1663, and ClsT are important for protecting against targocil during spore germination.

10.1128/mBio.03375-19.2 FIG S2 Zone of inhibition on LB agar plates surrounding discs loaded with the indicated compounds before growth of the B. anthracis Sterne (parental strain), Δ*edsRS*, and Δ*BAS1661-BAS1663*Δ*clsT* strains was observed. Data represent averages of results from two independent experiments ± standard deviations (SD). Download FIG S2, TIF file, 0.5 MB. Copyright © 2020 Laut et al. 2020 Laut et al. This content is distributed under the terms of the Creative Commons Attribution 4.0 International license.

### EdsRS activation of *clsT* expression is required to combat targocil-induced envelope permeability.

**FIG 5:**
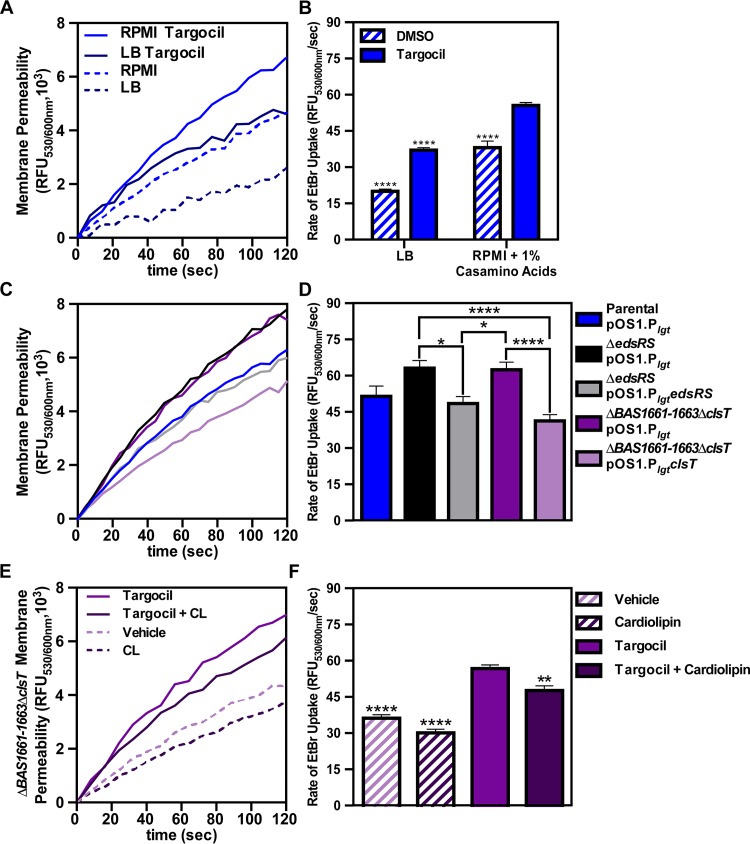
Cardiolipin synthesis is required for an intact cellular barrier during targocil treatment. (A) Ethidium bromide uptake over time after growth of B. anthracis Sterne (parental strain) in RPMI medium plus 1% Casamino Acids or LB with or without 100 μg/ml targocil. Data represent averages of results from three independent experiments performed in biological triplicate ± SEM. (B) The rate of ethidium bromide uptake shown in panel A. Data represent averages of results from three independent experiments performed in biological triplicate ± SEM. Statistical significance compared to parental strain was determined using a two-way ANOVA with a Tukey’s test adjustment for multiple comparisons (****, *P ≤ *0.0001). (C) Ethidium bromide uptake over time after growth of the parental strain, Δ*edsRS* strain, and Δ*BAS1661*–*BAS1663*Δ*clsT* strain containing either empty vector (pOS1 P*_lgt_*) or vectors for phenotypic complementation in RPMI medium plus 1% Casamino Acids with 100 μg/ml targocil. Data represent averages of results from three independent experiments performed in biological triplicate ± SEM. (B) The rate of ethidium bromide uptake shown in panel C. Data represent averages of results from three independent experiments performed in biological triplicate ± SEM. Statistical significance compared to the parental strain was determined using a one-way ANOVA with a Tukey’s test adjustment for multiple comparisons (*, *P ≤ *0.05; ****, *P ≤ *0.0001). (E) Ethidium bromide uptake over time after growth of strain Δ*BAS1661–BAS1663*Δ*clsT* in RPMI medium plus 1% Casamino Acids with or without 100 μg/ml targocil, in the presence and absence of 100 μg/ml cardiolipin (CL). Data represent averages of results from three independent experiments performed in biological quadruplicate ± SEM. (F) The rate of ethidium bromide uptake shown in panel E. Data represent averages of results from three independent experiments performed in biological quadruplicate ± SEM. Statistical significance compared to targocil-treated samples was determined using a one-way ANOVA with a Dunnett’s test adjustment for multiple comparisons (**, *P ≤ *0.01; ****, *P ≤ *0.0001).

ClsT is predicted to be a cardiolipin synthase. Cardiolipin is a component of phospholipid bilayers and is primarily associated with bacterial and mitochondrial membranes. Cardiolipin makes up roughly 17% of total phospholipid in pathogenic bacillus species and is mobilized as a component of the membrane damage response (49, 50). Due to the increased targocil sensitivity of the Δ*BAS1661-BAS1663*Δ*clsT* mutant during spore germination and outgrowth, upregulation of *clsT* in targocil treatment, and the role of cardiolipin in membrane maintenance, we hypothesized that targocil induces barrier damage in B. anthracis. The permeability of the cellular envelope can be quantified using an ethidium bromide (EtBr) uptake assay to measure the rate at which EtBr crosses the cell wall and lipid bilayer (51–53). Targocil induced permeability in the parent B. anthracis strain in both LB and RPMI medium plus Casamino Acids (Fig. 5A). However, the rate of EtBr uptake after targocil treatment was significantly higher in the cultures grown in semidefined media than in those grown in rich media (Fig. 5B). From these results, we conclude that targocil increases cellular permeability.

Next, we hypothesized that activation of expression of *BAS1661-BAS1663clsT* by EdsRS is required for the response to targocil-induced envelope damage. In the EtBr uptake assay, the Δ*BAS1661-BAS1663*Δ*clsT* and Δ*edsRS* strains exhibited increased barrier permeability relative to the parent strain after 30 min of exposure to 100 μg/ml targocil in RPMI medium (Fig. 5C). Constitutive expression of *edsRS* in the Δ*edsRS* background resulted in a significant decrease in the permeability of this mutant, reaching levels comparable to those seen with the parent strain (Fig. 5D). This indicates that EdsRS activation is important to protect against targocil-induced envelope permeability but does not exclude the possibility that activation occurs via direct interaction of targocil with EdsS in the membrane. Interestingly, the increased permeability of the Δ*BAS1661-BAS1663*Δ*clsT* strain was able to be complemented by expression in *trans* of the cardiolipin synthase gene *clsT.* In fact, overexpression of *clsT* led to a 35% reduction in envelope permeability compared to the Δ*BAS1661-BAS1663*Δ*clsT* strain in the presence of targocil (Fig. 5D). These results indicate that *edsRS* and *clsT* are required for preserving envelope integrity following targocil exposure.

The addition of exogenous cardiolipin to bacteria can alter the phenotypes observed with envelope-targeting antimicrobials (54–56). Therefore, cardiolipin was added to targocil-exposed Δ*BAS1661-BAS1663*Δ*clsT* cultures, which resulted in significantly reduced envelope permeability (Fig. 5E). The rates of EtBr uptake in the vehicle and cardiolipin-only control samples were comparable (Fig. 5F), suggesting that the effect of exogenous cardiolipin is realized only upon membrane damage. Overall, these data indicate that EdsRS and ClsT respond to targocil-mediated envelope damage in B. anthracis.

### EdsRS responds to targocil-induced envelope permeability via synthesis of cardiolipin.

**FIG 6:**
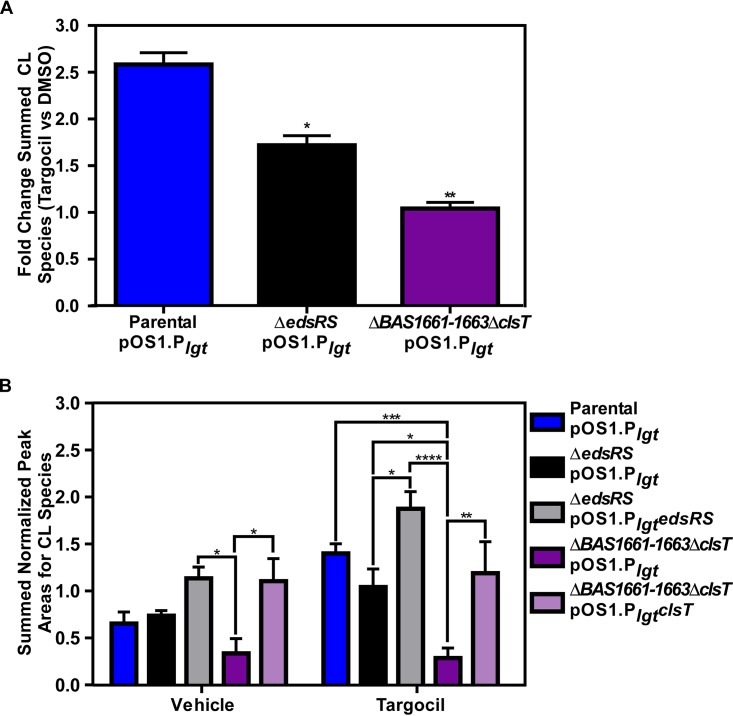
Targocil activation of EdsRS is required for ClsT-dependent increases in cardiolipin. (A) Whole-cell cardiolipin relative quantification using liquid chromatography coupled to tandem mass spectrometry (LC-MS/MS). Fold change of B. anthracis Sterne (parental strain), Δ*edsRS*, and Δ*BAS1661-BAS1663*Δ*clsT* strains grown in RPMI medium plus 1% Casamino Acids and then exposed to 100 μg/ml targocil was determined relative to vehicle-treated samples. Data represent averages of results from two technical runs performed in biological triplicate ± SEM. Statistical significance of results of comparisons of samples between treatment groups was determined using a two-way ANOVA with a Tukey’s test adjustment for multiple comparisons (*, *P ≤ *0.05; **, *P ≤ *0.01). (B) Cardiolipin relative quantification of complementation strains for the parental, Δ*edsRS*, and Δ*BAS1661–BAS1663*Δ*clsT* strains using LC-MS/MS. Cultures were also grown in RPMI medium plus 1% Casamino Acids and then exposed to 100 μg/ml targocil for 30 min. Data represent averages of results from two technical runs performed in biological triplicate ± SEM. Statistical significance compared to targocil-treated samples was determined using a two-way ANOVA with a Tukey’s test adjustment for multiple comparisons (*, *P ≤ *0.05; **, *P ≤ *0.01; ***, *P ≤ *0.001; ****, *P ≤ *0.0001).

EdsRS responds to targocil and increases expression of *clsT*, and this activation maintains the cell barrier in B. anthracis. Therefore, we hypothesized that cardiolipin levels within the bacterial membrane increase in the presence of targocil and that this is dependent on expression of both *edsRS* and *clsT.* To test this, liquid chromatography with tandem mass spectrometry (LC-MS/MS) was used to quantify relative levels of cardiolipin species in B. anthracis. Whole-cell lysates were collected after a 30-min exposure to 100 μg/ml targocil in RPMI medium plus Casamino Acids prior to lipid extraction and LC-MS/MS analysis (Fig. S3). Comparisons of cardiolipin levels in the parent strain with and without targocil exposure indicated that there was a significant increase in the level of cardiolipin after treatment (Fig. 6A). In support of this hypothesis, the Δ*edsRS* and Δ*BAS1661-BAS1663*Δ*clsT* strains did not show a significant increase in cardiolipin levels upon targocil treatment (Fig. 6A). In accordance with the idea of the importance of ClsT, the Δ*BAS1661-BAS1663*Δ*clsT* strain had a lower level of cardiolipin than the parent strain even in the absence of targocil. The phenotypes observed for the mutant strains were complemented above the parent strain levels when *edsRS* or *clsT* was constitutively expressed in *trans* (Fig. 6B). Consistent with earlier experiments (Fig. 5C and D), complementation of cardiolipin levels in the Δ*BAS1661-BAS1663*Δ*clsT* strain required only expression of the cardiolipin synthase (*clsT*). Therefore, the cellular response to targocil involves the synthesis of cardiolipin by ClsT. The expression of *clsT* under these conditions is driven by signaling through EdsRS, establishing this TCS as being required for sensing and responding to targocil-mediated envelope damage in B. anthracis.

10.1128/mBio.03375-19.3 FIG S3 (A) A mass spectrum from LC-MS/MS analysis of a parental B. anthracis lipid extract shows cardiolipin (CL) species differing in alkyl chain lengths and numbers of unsaturations from *m*/*z* 1,280 to 1,380. Three cardiolipin lipids are annotated with low mass error in parts per million (ppm) compared to the theoretical *m*/*z* value for the cardiolipin lipid. (B) The MS/MS spectra for annotated cardiolipin lipids presented in panel A show multiple fatty acid fragments that combine to form the total chain lengths and unsaturations for the cardiolipin species. Due to the presence of many cardiolipin isomers, various fatty acid fragments can be combined to construct the precursor cardiolipin. Download FIG S3, TIF file, 0.6 MB. Copyright © 2020 Laut et al. 2020 Laut et al. This content is distributed under the terms of the Creative Commons Attribution 4.0 International license.

## DISCUSSION

In this study, the B. anthracis response to the antimicrobial compound targocil was analyzed. Targocil induces damage to the cellular envelope (Fig. 5A and B) and activates the TCS EdsRS (Fig. 2B). Upon activation, EdsRS increases expression of a cardiolipin synthase (ClsT) (Fig. 3C). EdsRS and ClsT are required for the production of cardiolipin in response to targocil (Fig. 6) to protect B. anthracis from damage caused by targocil (Fig. 5C to E). Collectively, these data uncover a TCS that is activated to alter membrane composition in response to cell envelope damage.

Due to the synthetic nature of targocil, it is unlikely that EdsRS evolved to respond to this antimicrobial. However, the ability of B. anthracis to activate a TCS in response to envelope damage highlights the adaptability of this pathogen. The presence of over 40 TCSs equips B. anthracis to respond to a wide range of environments (57). Thus far, studies have assigned activating stimuli to 11 of these systems, but not all of them have defined downstream functions within the cell (58–64). Despite the importance of TCSs, there are still over 30 histidine kinase-response regulator pairs in B. anthracis that are not well understood. Our work exemplifies the importance of studying TCSs in this pathogen to uncover the contribution of signaling responses to anthrax pathogenesis.

The data presented here suggest that cardiolipin abundance in the membrane affects the efficacy of antimicrobials targeted to the cell envelope and that alterations in the regulation of cardiolipin synthesis represent a bacterial strategy for adaptation. B. anthracis encodes five cardiolipin synthase genes. This genetic redundancy in the biogenesis of cardiolipin has been reported for other bacterial species. Bacterial cardiolipin synthases (Cls) classically synthesize cardiolipin through the condensation of two molecules of phosphatidylglycerol to produce cardiolipin and glycerol (55, 65). The *cls* genes have been studied in Escherichia coli, which codes for three of these enzymes (ClsA, ClsB, and ClsC). Each of the Cls enzymes in this bacterium uses unique precursors and contributes to cardiolipin abundance under distinct conditions, including stationary or hyperosmotic growth (40, 66). This enables tight control of membrane phospholipid composition and preservation of resources. Similarly to E. coli, B. subtilis encodes three cardiolipin synthases (ClsA, YwjE, and YwiE) with differentially ascribed functions (67). ClsA is the primary cardiolipin synthase in this species during vegetative growth and osmotic stress but works together with YwjE to produce the phospholipid during the process of sporulation (67–69). Although it has not been empirically tested, transcription data suggest that the third synthase gene, *ywiE*, is involved in the response to heat shock (70).The use of multiple Cls variants is important in Gram-positive pathogens. The human pathogen S. aureus expresses two cardiolipin synthases (Cls1 and Cls2) (71, 72). Cls2 is the primary producer of cardiolipin, which accumulates in S. aureus during stationary-phase growth. However, after phagocytosis and during growth under high-salt conditions, both Cls1 and Cls2 contribute to cardiolipin synthesis (72). These examples highlight the versatility of bacterial phospholipid synthesis. Synthesis of cardiolipin depends on the growth phase and specific stress encountered and results in unique routes of biogenesis depending on available lipid resources. The extreme adaptability of B. anthracis to survive in the environment and during infection of mammalian hosts is likely supported not only by the presence of TCSs but also by flexibility in the pathways used for generation of key cellular molecules such as cardiolipin.

Cardiolipin has been associated with the efficacy of antimicrobials, specifically, daptomycin. Daptomycin forms pores in the membranes of bacteria, resulting in barrier permeabilization, and has been linked to disruption of cell division (73–75). Daptomycin was first used clinically in 2003, and since then, resistance to this drug has been reported in *Enterococcus* and *Staphylococcus* species (76). Sequencing of daptomycin-resistant *Enterococcus* strains identified mutations within a cardiolipin synthase (77, 78). Although the mechanism by which cardiolipin protects against daptomycin remains unclear, a follow-up study found that addition of exogenous cardiolipin to liposomes diminished the pore-forming activity of daptomycin (55). However, a transposon insertion sequencing (Tn-Seq) study in S. aureus found that mutation of a cardiolipin synthase conferred resistance to daptomycin (79). These results suggests that bacterial cells can increase the abundance of cardiolipin within their outer membranes to repair damage by antimicrobials that breach this barrier, similarly to the findings presented here in B. anthracis, but that the consequence of these modifications can be species specific.

While cardiolipin synthesis can enable bacteria to resist some antimicrobial compounds, the role of cardiolipin in the response to antimicrobials is context dependent. Cardiolipins can also increase the efficacy of other antimicrobials. For instance, the toxicity of an amphiphilic aminoglycoside derivative against Pseudomonas aeruginosa was enhanced with addition of cardiolipin to the culture conditions (56). The derivative altered the biophysical structure of lipid bilayers to cause increased permeability in a cardiolipin-dependent manner (80). In another example, the activity of plantazolicin was interrogated in B. anthracis (54). Plantazolicin inserts into the cellular envelope of bacilli and disrupts membrane potential, indicating damage to the bilayer. Of the species tested, B. anthracis was the only Gram-positive organism sensitive to this compound (81). That study observed that EdsRS (then referred to as BAS5200-BAS5201) was upregulated upon plantazolicin exposure, providing an additional link between EdsRS and damage to the envelope of B. anthracis (54). In an effort to understand the mechanism of plantazolicin toxicity, selection studies were performed looking for resistant mutants. Molohon et al. identified isolates that showed resistance phenotypes containing mutations within *BAS1662* and *BAS1663* and the promoter region of the *BAS1661-BAS1663clsT* operon. As a follow-up to this finding, they showed that the presence of exogenous cardiolipin increases the efficacy and membrane insertion of plantazolicin. Therefore, EdsRS is activated by treatment with plantazolicin; however, subsequent induction of *BAS1661-BAS1663clsT* and increase in cardiolipin levels are detrimental due to the mechanism of drug insertion. Using two small molecules that are similar in function but divergent in mechanism, the work of Molohon et al. and the results reported here support the idea of links between envelope disruption, EdsRS two-component system signaling, and cardiolipin synthesis.

The intracellular environment of host immune cells is a site for germination of B. anthracis spores during infection (82–87). The importance of EdsRS signaling to the germination and viability of B. anthracis during outgrowth in the presence of envelope-damaging agents indicates that this system could be used during dissemination in vertebrate hosts. TCSs are important for intracellular survival, where they enable a response to metal limitation or acidic environments (88, 89). Future studies are needed to test the role of EdsRS signaling in resistance to barrier attack and germination within phagocytes. The subsequent EdsRS-dependent induction of ClsT synthesis of cardiolipin may reverse barrier permeability to promote survival of bacilli (Fig. 3C, 4B, 5C to F, and 6). Cardiolipin synthesis is induced after phagocytosis occurs in S. aureus (72). The kinetics of this induction in S. aureus suggest that increased membrane cardiolipin levels are important for defense against later stages of immune attack following escape from the host phagocyte (72). Therefore, the bacteria may respond to recognition of the host environment to prepare for survival during infection. The precise role of cardiolipin synthesis in B. anthracis immune evasion should be elucidated in future studies as this suggests a role for EdsRS in defense against host attack. Reacting to host effectors in this manner may promote B. anthracis pathogenesis, providing significant insight into how this organism causes severe infections.

## MATERIALS AND METHODS

### Bacterial strains and growth conditions.

The bacterial strains (Table 1), plasmids (Table 2), and primers (Table 3) used in this study are listed in the indicated tables. Bacillus anthracis strain Sterne was used in all experiments under biosafety level 2 (BSL2) conditions (31). Cultures were streaked from glycerol freezer stocks on LB agar (LBA) plates and grown at 30°C for 16 h. LB was inoculated using a single colony from these plates. Cultures were grown at 30°C with shaking at 180 rpm and aeration for all overnight growth or for any period of growth over 8 h. For growth assays performed for 8 h or less, growth occurred at 37°C. As noted, experiments were performed in RPMI medium (Thermo Fisher Scientific) plus 1% (wt/vol) Casamino Acids to serve as a defined medium. Plasmids were constructed using E. coli DH5α or TOP10 strains. Plasmids were then moved from E. coli to B. anthracis after first transforming them into E. coli K1077 or S. aureus RN4220. Antibiotics concentrations used were carbenicillin at 50 μg/ml for E. coli (reporter and complementation vectors), chloramphenicol at 10 μg/ml for S. aureus and B. anthracis (reporter and complementation vectors), and kanamycin at 20 μg/ml in B. anthracis and 40 μg/ml in E. coli (genetic manipulation vector).

**TABLE 1:** Bacterial strains

Species	Genotype	Description	Reference(s) or source
B. anthracis strain Sterne	Wild type/parental	Wild-type/parental laboratory stock	31
B. anthracis strain Sterne	Δ*edsRS*	In-frame deletion of *BAS5200* and *BAS5201*	This study
B. anthracis strain Sterne	Δ*edsRS BAS5207*::*edsRS*	In-frame deletion of *BAS5200* and *BAS5201* and genomic complementation of *BAS5200* and *BAS5201* within *BAS5207*	This study
B. anthracis strain Sterne	Δ*BAS1661-BAS1663*Δ*clsT*	In-frame deletion of *BAS1661, BAS1662, BAS1663*, and *clsT*	This study
E. coli strain K1077	Wild type	Wild-type laboratory stock for cloning	90
S. aureus strain RN4220	Wild type	Wild-type laboratory stock for cloning	101, 102

**TABLE 2:** Plasmids

Plasmid	Description	Reference or source
pLM4	Allelic exchange vector for B. anthracis	92
pLM4.*edsRS*	Vector to delete *edsRS*	This study
pLM4.*5207*	Vector to integrate genes within *BAS5207*	This study
pLM4.*edsRScomp*	Vector to integrate *edsRS* within *BAS5207*	This study
pLM4.*BAS1661-BAS1663clsT*	Vector to delete *BAS1661-BAS1663clsT*	This study
pOS1.P*_lgt_*	Empty vector	92
pOS1.P*_eds_xylE*	*xylE* reporter vector	This study
pOS1.P*_lgt_edsRS*	*edsRS* complementation vector	This study
pOS1.P*_lgt_clsT*	*clsT* complementation vector	This study

**TABLE 3:** Primers

Primer	Sequence	Use
*edsRS* KO XmaI fwd	GCATGACCCGGGATAGAAGTTTTACGTACATTTCG	Mutagenesis
*edsRS* KO SOE-L	CTTCCTTGGTACCTTATACCGCTTCCTGTCTTTTTC	Mutagenesis
*edsRS* KO SOE-R	CGGTATAAGGTACCAAGGAAGTTTACCTATGAATGG	Mutagenesis
*edsRS* KO SacI rev	GCATGAGAGCTCGTGCATACTTACTCACCATCC	Mutagenesis
*5207* XmaI fwd	GCATGACCCGGGCTATGTAATTAGCTGGG	Chromosomal insertion
*5207* SOE-L	GCTAGCGCATGCGGTACCGTTACTTTACCATCCGCACC	Chromosomal insertion
*5207* SOE-R	GGTACCGCATGCGCTAGCGTTAAAGACTTGGAGCCTGG	Chromosomal insertion
*5207* KO SacI rev	GCATGAGAGCTCCTTCATTCGTATCTCTATTAACG	Chromosomal insertion
*edsRScomp* KpnI fwd	GCATGAGGTACCTCGAATATATATCTTACCCG	Chromosomal complementation
*edsRScomp* SOE-L	CTTCTCTATCATCTCTCCACCCCCGCT	Chromosomal complementation
*edsRScomp* SOE-R	GGTGGAGAGATGATAGAGAAGAAGAGGATTG	Chromosomal complementation
*edsRScomp* NheI rev	GCATGAGAATTCGCTAGCGGACTATGTAACTAAGACGG	Chromosomal complementation
*BAS1661-BAS1663clsT* KO XmaI fwd	GCATGACCCGGGAAAGATATGCAGCGACTTACG	Mutagenesis
*BAS1661-BAS1663clsT* KO SOE-L	CAACCTTTATACCCCTATTTCGGTTTCTCTGC	Mutagenesis
*BAS1661-BAS1663clsT* KO SOE-R	CGAAATAGGGGTATAAAGGTTGATGGGAAAAGG	Mutagenesis
*BAS1661-BAS1663clsT* KO SacI rev	GCATGAGAGCTCATGAGGCCATATAAACGTGTC	Mutagenesis
P*_eds_xylE* EcoRI fwd	GCATGAGAATTCTATCTTACCCGATTGTATCATG	Reporter
P*_eds_xylE* SOE-L	CTTTGTTCATCTCTCCACCCCCGCTG	Reporter
P*_eds_xylE* SOE-R	GGGTGGAGAGATGAACAAAGGTGTAATGCGAC	Reporter
P*_eds_xylE* BamHI rev	GCATGAGGATCCTCAGGTCAGCACGGTCATG	Reporter
P*_lgt_edsRS* fwd	CAATTGAGGTGAACATATGCTCGAGATGATAGAGAAGAAGAGGATTGAGATTTTTC	Complementation
P*_lgt_edsRS* rev	AAACACTACCCCCTTGTTTGGATCCTATCCATCCCTTTTCTTCC	Complementation
P*_lgt_clsT* fwd	CAATTGAGGTGAACATATGCTCGAGATGAACATGATTAAAAAAATATTGC	Complementation
P*_lgt_clsT* rev	AAACACTACCCCCTTGTTTGGATCCTTATAAATAAAAATCCACCATCC	Complementation
*edsR* fwd	TTTTGCTCCTGCCATAAGCC	qRT-PCR
*edsR* rev	CGCCCTGGATACTTTGAACG	qRT-PCR
*edsS* fwd	CCCTCACCCCATCTTTTCCT	qRT-PCR
*edsS* rev	ATGGAAATGGCATTCGTGGT	qRT-PCR
*BAS1661* fwd	GCAAGAGTGGAGGAAGCACT	qRT-PCR
*BAS1661* rev	CCAACTGTCGGCTCATCCAT	qRT-PCR
*BAS1662* fwd	CAATGTAGCGGCCGAAGTTG	qRT-PCR
*BAS1662* rev	TCGTCGTCATTACAACCGCA	qRT-PCR
*BAS1663* fwd	GTAAAGTGGCAGGGACGGAT	qRT-PCR
*BAS1663* rev	TCCTGATAAGTTCGCTGCTGA	qRT-PCR
*clsT* fwd	GAAGCGGCGTATCCATACAT	qRT-PCR
*clsT* rev	GCCCTACAATAGGACCACCA	qRT-PCR

### Preparation of compound stocks.

Carbenicillin stocks (50 mg/ml) were prepared in H_2_O and stored at –20°C. Chloramphenicol stocks (10 mg/ml) were made in 70% EtOH and stored at –20°C. Targocil stocks (10 mg/ml) were made in dimethyl sulfoxide (DMSO) and stored at –20°C. A stock of cardiolipin sodium salt (10 mg/ml) was made in 100% EtOH and stored at –20°C. All chemicals were purchased from Sigma-Aldrich unless otherwise noted.

### Genetic manipulation of B. anthracis.

Genetic manipulation was performed as previously described (58, 59). Electroporations were performed with modifications as described previously (58, 59, 90). The generation of knockout strains was performed by inserting the flanking sequences for genes of interest in the mutagenesis plasmid pLM4. Briefly, flanking sequences were amplified using a distal primer containing a restriction enzyme site and a proximal primer containing a short sequence overlapping the adjacent flanking region. PCR-amplified DNA was fused using PCR sequence overlap extension (PCR-SOE) as described previously (91). *BAS5207* was selected as a B. anthracis strain Sterne pseudogene based on NCBI RefSeq annotation (GenBank genomic sequence accession no. NC_005945). In other strains, including B. cereus ATCC 14597 and B. thuringiensis BMB181, *BAS5207* orthologues are annotated as encoding predicted LPXTG cell wall anchor domain-containing proteins with homology to collagen adhesion proteins. However, in strain Sterne, *BAS5207* is interrupted by two frameshift mutations (a single-base-pair insertion corresponding to nucleotide 1805 and a 14-bp insertion between nucleotides 5040 and 5041 in the *BAS5207* open reading frame [ORF]) and multiple deletions at the C terminus, leading us to conclude that *BAS5207* is likely a pseudogene in this strain. To construct a chromosomal insertion of *edsRS* into the pseudogene locus *BAS5207*, plasmid pLM4.*5207* was constructed as described above with the exception that the flanking region overlap site contained recognition sequences for the restriction nucleases NheI and KpnI. The *edsRS* genes were fused to the *eds* promoter by PCR-SOE, and the resulting product was inserted between the NheI and KpnI sites of pLM4.*5207* to generate a plasmid for chromosomal complementation of *edsRS*, pLM4.*edsRScomp*. Mutagenesis was performed using this vector as described previously (92) and confirmed by PCR and Sanger sequencing.

### Expression studies.

B. anthracis cultures were grown in LB at 37°C with shaking for 6 h (mid-log phase). Cultures were divided in half and then dosed with either DMSO control or 10 μg/ml targocil. Cultures were returned to 37°C for 10 min. Cultures were mixed at a 1:1 ratio with a 1:1 mixture of cold acetone and EtOH and stored at –80°C until RNA isolation. RNA was isolated using an RNeasy kit (Qiagen), and DNA was removed using a Turbo DNA-free kit (Invitrogen, Thermo Scientific). Quantification of RNA was performed using a Thermo Scientific NanoDrop spectrophotometer, and then cDNA was synthesized using an iScript cDNA synthesis kit (Bio-Rad). RNA sequencing was performed by HudsonAlpha as described previously (93). qRT-PCR was performed as previously described using the threshold cycle (ΔΔ^−^*^CT^*) method and iQ SYBR green Supermix (Bio-Rad) (94).

### Targocil toxicity growth curves.

Strains of interest were streaked on LBA and grown at 30°C for 16 to 18 h. Single colonies were used to start cultures containing the same medium used for the assay as annotated and were grown for 16 h at 30°C with shaking. Cultures were diluted 1:100 into fresh media and grown with shaking for 6 h at 37°C. One microliter of each culture was added to 99 μl of LB or RPMI medium plus 1% Casamino Acids, as annotated, containing 0 to 100 μg/ml targocil in a 96-well flat-bottomed plate. Growth was monitored over time at 37°C by measuring the optical density at 600 nm in a BioTek Epoch2 spectrophotometer and analyzed with BioTek Gen5 software.

### XylE assay.

To monitor *eds* promoter activity, a XylE reporter plasmid was generated. In the pOS1 vector (95), the *eds* promoter (P*_eds_*) sequence was fused to *xylE* using PCR-SOE as described previously (96). This vector was carried through the cloning strains described above followed by electroporation into B. anthracis. Strains containing the P*_eds_xylE* reporter plasmid were grown at 37°C in LB containing chloramphenicol and 0 to 1 μg/ml targocil. After 6 h, the abundance of the XylE enzyme present in B. anthracis cellular lysates was assessed by measuring the rate at which catechol was converted to 2-hydroxymuconic acid using a spectrophotometer as described previously (58).

### Spore preparation.

Modified G medium (MGM) sporulation media were used for spore preparation (97). A single colony of B. anthracis was used to inoculate LB and grown for 4 h at 37°C with shaking. This culture was then back-diluted 1:20 into a flask containing MGM at a volume that provides maximum aeration. Cultures were then grown at 37°C for 72 h. The bacterial pellet was collected using centrifugation and washed repeatedly using sterile deionized water (diH_2_O). After washing of the spores was performed a minimum of 4 times, the culture suspension was incubated at 65°C for 30 min. Samples were washed with sterile diH_2_O again and then diluted and plated onto LB for quantification.

### Spore outgrowth curves.

Spores were prepared as described above. Enumerated spores were diluted to a concentration of 1 × 10^8^ using diH_2_O. One microliter of each spore preparation was added to 99 μl media containing 0 or 25 μg/ml targocil in a 96-well flat-bottomed plate. Growth was monitored over time at 37°C by measuring the optical density at 600 nm in a BioTek Epoch2 spectrophotometer and analyzed with BioTek Gen5 software.

### Quantification of spore germination.

Spores were prepared as described above. Enumerated spores were diluted to a concentration of 1 × 10^6^ in LB broth containing a vehicle control or 100 μg/ml targocil. Cultures were incubated at 37°C for 5 min. Samples were dilution plated onto LB agar to determine the total bacterial density. The same samples were then incubated at 65°C for 30 min to lyse vegetative cells. The boiled suspension was dilution plated to enumerate spore counts.

### Disc diffusion assay.

This assay was modified from studies previously described (98). Overnight cultures were grown at 30°C for 16 h. Following incubation, bacteria were mixed with top agar made from LB broth and poured onto LB agar plates. Sterile discs were placed onto the plates and loaded with one of each of the compounds included in our screening panel. The plates were incubated at 30°C for 18 h and then imaged. The diameter of the zone of inhibition was then measured. Experiments were performed twice.

### Envelope permeability assay.

Overnight cultures were grown at 30°C in RPMI medium plus 1% Casamino Acids with chloramphenicol. Cultures were normalized to an optical density at 600 nm (OD_600_) of 0.3 in fresh media using a black-walled 96-well plate. The indicated treatments were added to the cultures and allowed to incubate at 37°C for 30 min. Ethidium bromide was added rapidly at 1 μg/ml, and the fluorescence (excitation [Ex], 530 nm; emission [Em], 600 nm) was immediately read kinetically using a BioTek Cytation 5 plate reader.

### Cardiolipin relative quantification.

Lipids were extracted from cell pellets (normalized by OD) using the Bligh-Dyer method (99). Extracts were dried under nitrogen and reconstituted in 100 μl of 65% acetonitrile–30% isopropyl alcohol–5% water. All samples contained 5 μg/ml of a cardiolipin standard [CL(16:0/18:1)] (Avanti Polar Lipids, Inc., Alabaster, AL, USA). A 15-μl volume of each sample was injected into an Acquity Arc ultra-high-performance liquid chromatography (UPLC) system (Waters Corporation, Milford, MA, USA). Lipids were separated using an Acquity UPLC HSS C_18_ column (Waters Corporation, Milford, MA, USA) with 1.8-μm particle size and dimensions of 2.1 mm by 150 mm. The aqueous solvent system (solvent A) consisted of 60% acetonitrile, 40% water, 0.1% formic acid, and 10 mM ammonium acetate. The organic solvent system (solvent B) consisted of 90% isopropyl alcohol, 10% acetonitrile, 10 mM ammonium acetate, and 0.1% formic acid (100). The following gradient was used: 0 min, 70% solvent A; 0 to 5 min, 70% to 57% solvent A; 5 to 5.1 min, 57% to 50% solvent A; 5.1 to 14 min, 50% to 30% solvent A; 14 to 21 min, 30% to 1% solvent A; 21 to 30 min, 1% solvent A; 30 to 30.1 min, 1% to 70% A. The column was allowed to equilibrate at 70% solvent A for 9.9 min prior to the next injection. The column heater was set at 40°C, and the flow rate was 0.22 ml/min. After separation, samples were introduced by electrospray ionization (2.5-kV capillary; 100°C source temperature; 40-V sampling cone) into a quadrupole-time of flight mass spectrometer (Waters Synapt G2-Si; Waters Corporation, Milford, MA, USA) for analysis in negative-ionization mode (trap and transfer collision energies, 15 V; resolution mode; ion mobility not enabled). Samples were analyzed in data-dependent mode with a survey window at mass-to-charge (*m*/*z*) ratios of 500 to 1,750 with a scan time of 0.2 s. Fragmentation data were acquired using a collision energy ramp of 6 to 147 eV (depending on the *m*/*z* value selected) with a 30-s exclusion window. The instrument was calibrated using sodium formate prior to analysis, and a lock spray containing a tuned mix of known *m*/*z* values (Agilent Technologies, Inc., Santa Clara, CA, USA) was infused (flow rate, 5 μl/min; scan time, 1 s; 10-s intervals; 3 scans averaged) during analysis for internal calibration of data postacquisition. Cardiolipin lipids eluted between 24 and 26 min. Data were analyzed by separately extracting ion chromatograms for all annotated cardiolipins within spectra obtained from parent strain samples. Peaks from extracted ion chromatograms were integrated manually and normalized to the area of the internal cardiolipin standard. Normalized areas were then summed for all annotated species. Data represent results from biological triplicates with two technical replicates each.

### Data availability.

RNA sequencing data were deposited in the Gene Expression Omnibus (GSE142363).
