# Supplementary material for: Bacillus anthracis Responds to Targocil-Induced Envelope Damage through EdsRS Activation of Cardiolipin Synthesis
Source: mBio. 2020 Mar 31;11(2):e03375-19. doi: 10.1128/mBio.03375-19 (PMC7157781; doi:10.1128/mBio.03375-19)
Supplement: TABLE S1 [file mBio.03375-19-st001.docx]

**Supplementary Table 1: Parental targocil vs. parental DMSO**

| **Locus** | **Log2 (Fold Change)** | **Corrected p-value ( Z Test )** |
| --- | --- | --- |
| BAS0298 | 2.66113 | 0 |
| BAS0299 | 3.3181753 | 0 |
| BAS0300 | 4.7890596 | 0 |
| BAS0301 | 4.6925144 | 0 |
| BAS0302 | 4.761792 | 0 |
| BAS0303 | 4.8750496 | 0 |
| BAS0304 | 3.6219492 | 0 |
| BAS0375 | 1.341074 | 0 |
| BAS0376 | 2.4744802 | 0 |
| BAS0447 | -2.216065 | 2.25E-03 |
| BAS0448 | -1.37328 | 1.50E-03 |
| BAS0629 | 5.290226 | 0 |
| BAS0630 | 5.145034 | 0 |
| BAS0900 | 2.4476304 | 0 |
| BAS0901 | 2.4229412 | 0 |
| BAS0902 | 2.405727 | 0 |
| BAS0903 | 1.7964697 | 0 |
| BAS0959 | 1.0817976 | 0 |
| BAS0961 | 1.8198357 | 0 |
| BAS1320 | 1.0020113 | 0 |
| BAS1391 | -2.5997314 | 0 |
| BAS1661 | 2.8152132 | 0 |
| BAS1662 | 2.5660582 | 0 |
| BAS1663 | 2.4968348 | 0 |
| BAS1664 | 2.6609936 | 0 |
| BAS1889 | -1.0443525 | 0 |
| BAS2164 | -1.166723 | 6.29E-05 |
| BAS3022 | 1.2139626 | 0 |
| BAS3023 | 1.1851959 | 0 |
| BAS3300 | 1.3857727 | 0 |
| BAS3384 | 1.2540779 | 0 |
| BAS3386 | 2.4372015 | 0 |
| BAS3387 | 2.378869 | 0 |
| BAS3388 | 2.4362726 | 0 |
| BAS3403 | -2.1725755 | 6.90E-05 |
| BAS3467 | 1.0735712 | 0 |
| BAS3910 | 1.8114672 | 0 |
| BAS4018 | -1.2304945 | 0 |
| BAS4019 | -1.4040194 | 0 |
| BAS4020 | -1.2432327 | 0 |
| BAS4021 | -1.1240253 | 0 |
| BAS4464 | 4.458848 | 0 |
| BAS4465 | 4.393877 | 0 |
| BAS4734 | -1.5486361 | 1.13E-02 |
| BAS4945 | 1.0680733 | 0 |
| BAS5200 | 7.559718 | 0 |
| BAS5201 | 7.967046 | 0 |
| BAS5202 | 11.675568 | 0 |
| BAS5203 | 11.414888 | 0 |
| BAS5288 | 2.5798273 | 0 |
| BAS5289 | 5.30478 | 0 |
