# Supplementary material for: Bacillus anthracis Responds to Targocil-Induced Envelope Damage through EdsRS Activation of Cardiolipin Synthesis
Source: mBio. 2020 Mar 31;11(2):e03375-19. doi: 10.1128/mBio.03375-19 (PMC7157781; doi:10.1128/mBio.03375-19)
Supplement: TABLE S2 [file mBio.03375-19-st002.docx]

**Supplementary Table 2: Δ*edsRS* targocil vs. ΔedsRS DMSO**

| **Locus** | **Log2 (Fold Change)** | **Corrected p-value ( Z Test )** |
| --- | --- | --- |
| BAS0225 | -1.9936426 | 8.25E-04 |
| BAS0383 | -1.0841188 | 0 |
| BAS0422 | -1.2937973 | 9.65E-05 |
| BAS0447 | -1.6582851 | 5.78E-03 |
| BAS0739 | -1.1795282 | 1.50E-13 |
| BAS0792 | -1.0312767 | 1.67E-15 |
| BAS1391 | -2.5955887 | 0 |
| BAS1889 | -1.018156 | 0 |
| BAS2179 | -1.4067283 | 4.76E-04 |
| BAS3088 | -1.2082615 | 5.93E-07 |
| BAS3375 | -1.4148128 | 1.14E-06 |
| BAS3522 | -1.2061193 | 9.94E-05 |
| BAS3544 | -1.6680828 | 2.06E-04 |
| BAS3655 | -2.0111318 | 3.81E-10 |
| BAS4018 | -1.2382097 | 0 |
| BAS4019 | -1.3210001 | 0 |
| BAS4020 | -1.2279987 | 0 |
| BAS4021 | -1.1596413 | 0 |
| BAS4625 | -1.4263597 | 1.54E-04 |
| BAS4719 | -1.5832007 | 6.22E-03 |
| BAS4910 | -1.2719345 | 1.72E-07 |
| BAS5102 | -1.1454148 | 1.97E-05 |
| BAS5200 | -1.0037732 | 3.30E-03 |
| BAS0298 | 2.6809492 | 0 |
| BAS0299 | 3.3839474 | 0 |
| BAS0300 | 4.7595015 | 0 |
| BAS0301 | 4.8857727 | 0 |
| BAS0302 | 4.8181515 | 0 |
| BAS0303 | 4.858917 | 0 |
| BAS0304 | 3.7033749 | 0 |
| BAS0375 | 1.2661839 | 0 |
| BAS0376 | 2.0624151 | 0 |
| BAS0629 | 5.2277946 | 0 |
| BAS0630 | 5.102483 | 0 |
| BAS0655 | 1.1688995 | 0 |
| BAS0882 | 1.527105 | 2.58E-02 |
| BAS0900 | 2.1965241 | 0 |
| BAS0901 | 2.1561937 | 0 |
| BAS0902 | 2.0542974 | 0 |
| BAS0903 | 1.3169293 | 0 |
| BAS0959 | 1.0824289 | 0 |
| BAS0961 | 1.9214096 | 0 |
| BAS2825 | 1.0545716 | 0 |
| BAS3300 | 1.5477171 | 0 |
| BAS3384 | 1.1557169 | 3.88E-14 |
| BAS3386 | 2.400652 | 0 |
| BAS3387 | 2.4442577 | 0 |
| BAS3388 | 2.3708296 | 0 |
| BAS3910 | 1.8889132 | 0 |
| BAS4464 | 4.1995325 | 0 |
| BAS4465 | 4.043395 | 0 |
| BAS4945 | 1.1565027 | 0 |
| BAS5288 | 2.6898975 | 0 |
| BAS5289 | 5.2167654 | 0 |
