# Supplementary material for: Bacillus anthracis Responds to Targocil-Induced Envelope Damage through EdsRS Activation of Cardiolipin Synthesis
Source: mBio. 2020 Mar 31;11(2):e03375-19. doi: 10.1128/mBio.03375-19 (PMC7157781; doi:10.1128/mBio.03375-19)
Supplement: TABLE S3 [file mBio.03375-19-st003.docx]

**Supplementary Table 3: Δ*edsRS* targocil vs. parental targocil**

| **Locus** | **Log2 (Fold Change)** | **Corrected p-value ( Z Test )** |
| --- | --- | --- |
| BAS1661 | -2.8863726 | 0 |
| BAS1662 | -2.5855646 | 0 |
| BAS1663 | -2.3593788 | 0 |
| BAS1664 | -2.3234634 | 0 |
| BAS1957 | 1.074918 | 3.44E-02 |
| BAS3522 | -1.1107314 | 1.11E-03 |
| BAS3544 | -1.2132076 | 3.43E-02 |
| BAS3655 | -1.8650764 | 1.56E-04 |
| BAS4734 | 1.5793983 | 3.43E-02 |
| BAS4910 | -1.1477416 | 7.08E-03 |
| BAS5064 | -1.120647 | 1.27E-03 |
| BAS5200 | -14.825722 | 0 |
| BAS5201 | -14.311115 | 0 |
| BAS5202 | -11.143267 | 0 |
| BAS5203 | -11.659903 | 0 |
