# Supplementary material for: Bacillus anthracis Responds to Targocil-Induced Envelope Damage through EdsRS Activation of Cardiolipin Synthesis
Source: mBio. 2020 Mar 31;11(2):e03375-19. doi: 10.1128/mBio.03375-19 (PMC7157781; doi:10.1128/mBio.03375-19)
Supplement: TABLE S4 [file mBio.03375-19-st004.docx]

**Supplementary Table 4: Δ*edsRS* DMSO vs. parental DMSO**

| **Locus** | **Log2 (Fold Change)** | **Corrected p-value ( Z Test )** |
| --- | --- | --- |
| BAS2678 | -1.7751769 | 2.26E-02 |
| BAS4458 | -1.4728799 | 5.19E-03 |
| BAS5200 | -6.2622304 | 0 |
| BAS5201 | -5.85716 | 0 |
| BAS0426 | 1.2504287 | 2.82E-03 |
| BAS3022 | 1.2801132 | 0 |
| BAS3023 | 1.1470747 | 0 |
| BAS4678 | 1.283607 | 3.76E-04 |
| BAS5102 | 1.2072129 | 1.96E-05 |
| BAS5211 | 1.0172274 | 2.82E-03 |
