# Supplementary figures and images for: Bacillus anthracis Responds to Targocil-Induced Envelope Damage through EdsRS Activation of Cardiolipin Synthesis
Source: mBio. 2020 Mar 31;11(2):e03375-19. doi: 10.1128/mBio.03375-19 (PMC7157781; doi:10.1128/mBio.03375-19)

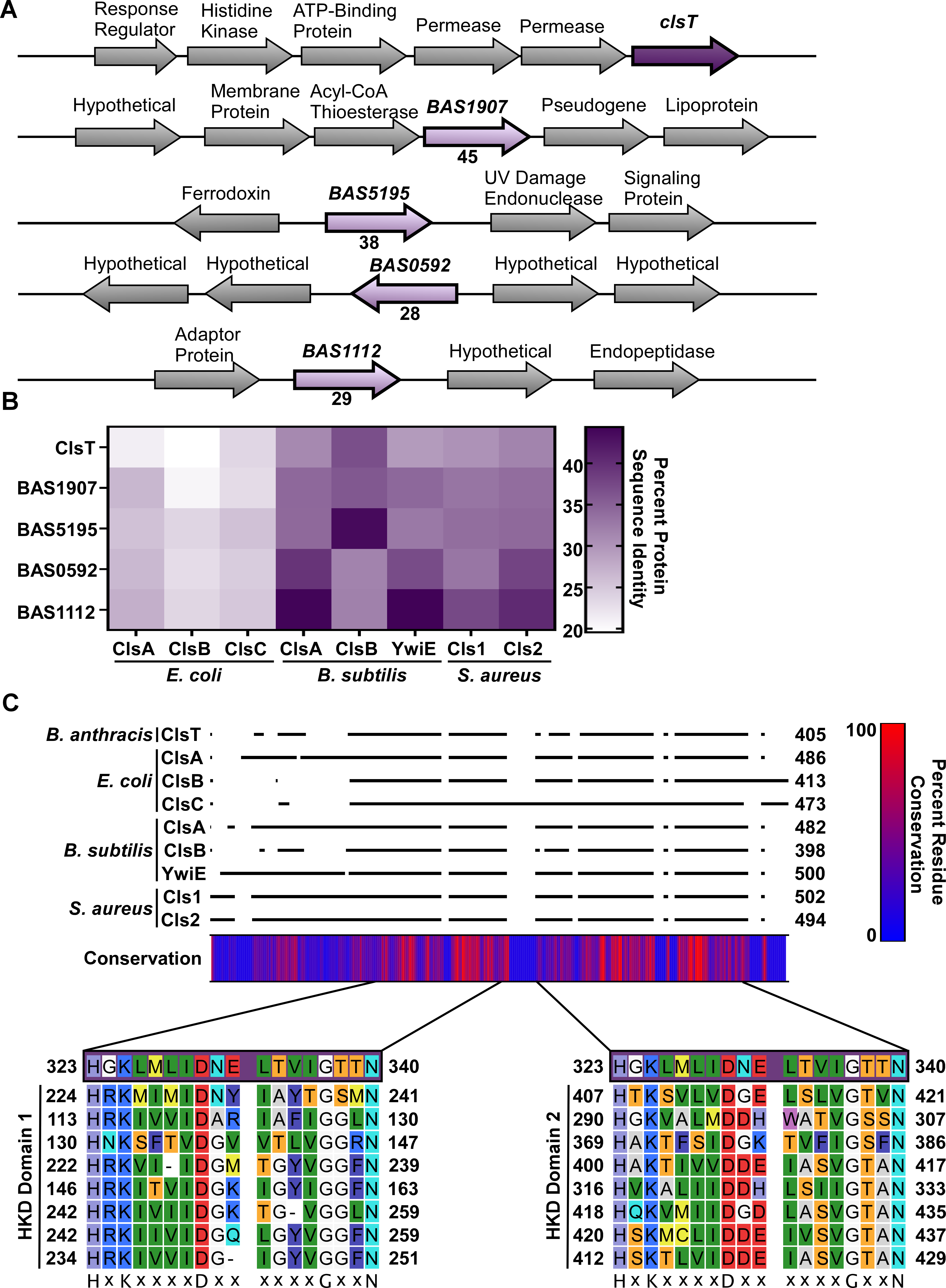

Supplement: FIG S1 [file mBio.03375-19-sf001.tif]

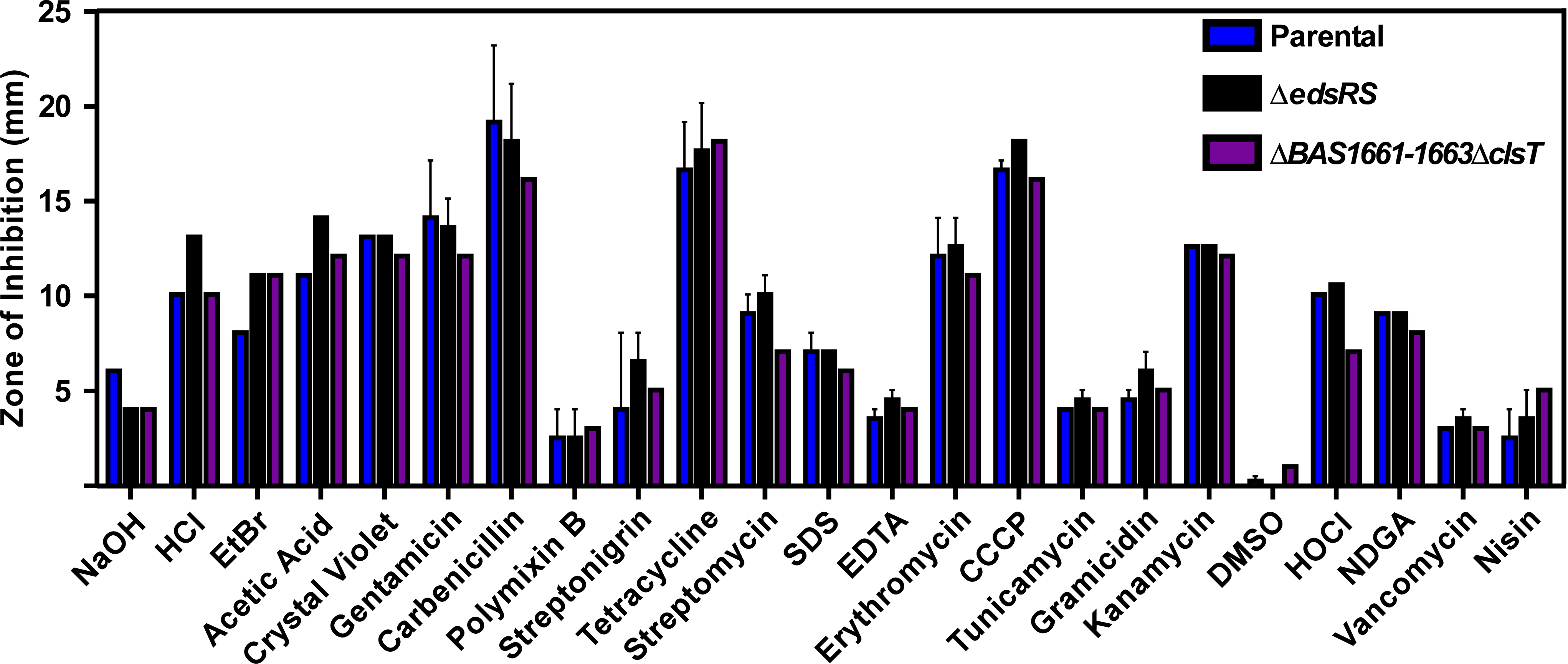

Supplement: FIG S2 [file mBio.03375-19-sf002.tif]

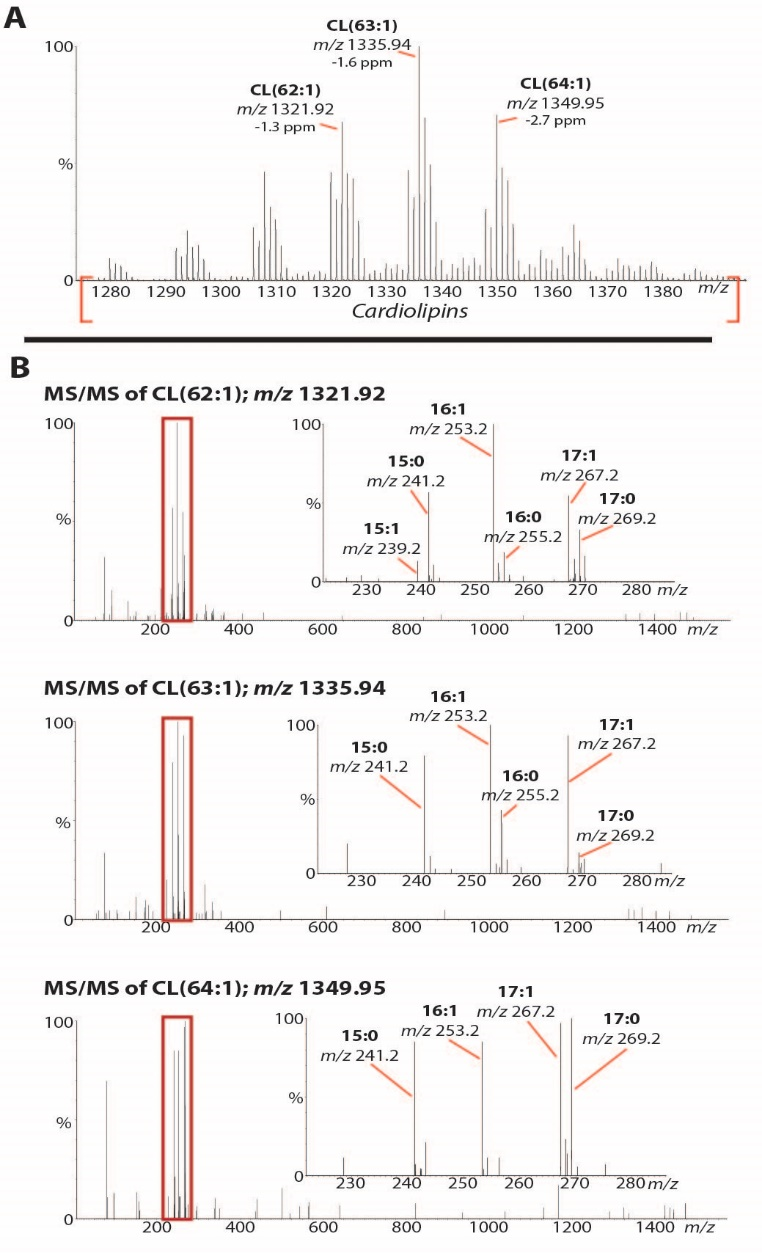

Supplement: FIG S3 [file mBio.03375-19-sf003.tif]
